# Evaluation of Winter Ticks (Dermacentor albipictus) Collected from North American Elk (Cervus canadensis) in an Area of Chronic Wasting Disease Endemicity for Evidence of PrP^CWD^ Amplification Using Real-Time Quaking-Induced Conversion Assay

**DOI:** 10.1128/mSphere.00515-21

**Published:** 2021-08-04

**Authors:** N. J. Haley, D. M. Henderson, K. Senior, M. Miller, R. Donner

**Affiliations:** a Department of Microbiology and Immunology, College of Graduate Studies, Midwestern University, Glendale, Arizona, USA; b CWDEvolution LLC, San Diego, California, USA; Colorado State University

**Keywords:** prion, elk, chronic wasting disease, tick, RT-QuIC, tick-borne pathogens

## Abstract

Chronic wasting disease (CWD) is a progressive and fatal spongiform encephalopathy of deer and elk species, caused by a misfolded variant of the normal prion protein. Horizontal transmission of the misfolded CWD prion between animals is thought to occur through shedding in saliva and other forms of excreta. The role of blood in CWD transmission is less clear, though infectivity has been demonstrated in various blood fractions. Blood-feeding insects, including ticks, are known vectors for a range of bacterial and viral infections in animals and humans, though to date, there has been no evidence for their involvement in prion disease transmission. In the present study, we evaluated winter ticks (Dermacentor albipictus) collected from 136 North American elk (Cervus canadensis) in an area where CWD is endemic for evidence of CWD prion amplification using the real-time quaking-induced conversion assay (RT-QuIC). Although 30 elk were found to be CWD positive (22%) postmortem, amplifiable prions were found in just a single tick collected from an elk in advanced stages of CWD infection, with some evidence for prions in ticks collected from elk in mid-stage infection. These findings suggest that further investigation of ticks as reservoirs for prion disease may be warranted.

**IMPORTANCE** This study reports the first finding of detectable levels of prions linked to chronic wasting disease in a tick collected from a clinically infected elk. Using the real-time quaking-induced conversion assay (RT-QuIC), “suspect” samples were also identified; these suspect ticks were more likely to have been collected from CWD-positive elk, though suspect amplification was also observed in ticks collected from CWD-negative elk. Observed levels were at the lower end of our detection limits, though our findings suggest that additional research evaluating ticks collected from animals in late-stage disease may be warranted to further evaluate the role of ticks as potential vectors of chronic wasting disease.

## INTRODUCTION

Chronic wasting disease (CWD) is a progressive and ultimately fatal neurodegenerative disease in cervids, including whitetail deer (Odocoileus virginianus), mule deer (Odocoileus hemionus), and North American elk (wapiti, Cervus canadensis) (1, 2). Like other diseases in this category—the transmissible spongiform encephalopathies or TSEs—CWD is caused by an infectious, misfolded variant of the normal cellular prion protein (PrP^C^) that is often designated PrP^CWD^ (3, 4). First identified in captive mule deer in northern Colorado and southern Wyoming in the late 1960s, CWD has now been reported in farmed and/or free-ranging cervids in 26 U.S. states, three Canadian provinces, South Korea, Norway, Sweden, and Finland (5–9).

*In vivo* studies, using both deer and mouse bioassay, and *in vitro* amplification assays have identified infectious prions or PrP^CWD^ in various bodily fluids and other forms of excreta, including saliva, blood, urine, and feces (10–19). In nature, it is thought that saliva is an important means of direct animal-to-animal transmission, while urine, feces, and decomposing carcasses may all play important roles in environmental contamination and subsequent exposure and transmission (2, 20, 21). The role of blood in the transmission of CWD and other prion diseases is less clear, though various blood fractions have been found to convey prion infection both in experimental studies in animal models and through rare natural infections in humans (11, 22–24). Importantly, the role of blood-feeding insects, including ticks, as vectors for prion transmission in nature has not been extensively evaluated.

Winter ticks (Dermacentor albipictus) are common external parasites found on moose, caribou, elk, and other large herbivores and are widely distributed across North America, Mexico, and Central America (25). They may be found in very high densities on moose, sometimes numbering in the tens of thousands on a single animal, and have been linked to declining moose populations across North America (26). Winter ticks are considered a “one-host” tick, taking several blood meals from a single host while progressing through larval and nymph stages to adulthood, though they are likely to seek a new host if dislodged prematurely from their primary host (27, 28). As a single-host tick, they have only rarely been implicated as vectors of disease, notably hemoparasites such as Anaplasma marginale and Babesia duncani (29, 30). Paradoxically, this one-host life cycle makes them ideal for evaluating tick species as potential reservoirs for CWD and other prions, as every blood meal over the course of their development is putatively taken from a single host, thus amplifying their exposure to the agent.

As part of a prior study evaluating the practicality of antemortem rectal biopsy specimen testing in managing CWD in ranched elk (31), we collected adult winter ticks from elk in an area with a high prevalence of CWD. Of the 136 elk sampled, 30 were found to be CWD positive either antemortem or postmortem (22%), at various stages of disease based on the diverse appearance of PrP^CWD^ in diagnostic tissues and apparent clinical symptoms suggestive of late-stage infection. In the present study, we evaluated these ticks in a blind manner using the real-time quaking-induced conversion assay (RT-QuIC), an assay used widely to amplify PrP^CWD^ in a range of substrates, including rectal biopsy specimens in the parent study (14–17, 31–36). We hypothesized that amplifiable PrP^CWD^ would be detected in ticks collected from CWD-positive elk, with ticks from elk in more advanced stages of disease having higher rates of amplification. We found PrP^CWD^ amplification in a single tick collected from an elk in an advanced stage of CWD, with evidence for amplifiable prions in ticks from elk in mid-stage CWD. Though the biological relevance of our findings remains unknown, they suggest that winter ticks, and potentially other tick species, could serve as low-level reservoirs for CWD in nature.

## RESULTS

### Summary of source elk testing.

A total of 30 of the 136 elk providing ticks for the present study were identified as CWD positive via postmortem immunohistochemistry of retropharyngeal lymph node (RLN) and obex tissue (22%). Based on the distribution of PrP^CWD^ in these tissues as well as clinical presentation, infected animals were estimated to be in various stages of disease from early and preclinical to late stage and symptomatic. A breakdown of estimated disease stages can be found in Table 1.

**TABLE 1:** Summary of elk providing tick samples for the present study

CWD status	Total no. of elk	Mean age (yr) (range)	No. of elk with the following Prnp genotype:^*a*^			Estimated disease stage^*b*^		
						Early	Middle	Late
			132MM	132ML	132LL	B or RLN (+)	B and RLN (+)	B and RLN (+) and symptomatic
Positive	30	5.2 (2–14)	18	10	2	5	24	1
Negative	106	3.07 (1–13)	41	49	16			

a Genotype is based on the amino acid coded for at position 132 of the elk prion gene, Prnp. Animals with a 132MM genotype are comparatively more susceptible, while those with a 132ML or 132LL genotype are less likely to be found CWD positive in natural and experimental conditions.

b Disease stage estimates are based on immunohistochemical detection of PrP^CWD^ in the elk’s brain (B) and/or retropharyngeal lymph node (RLN) and clinical signs consistent with late-stage infection, including behavior abnormalities and poor body condition scores.

### Optimized dilution of ticks for use in RT-QuIC.

**FIG 1:**
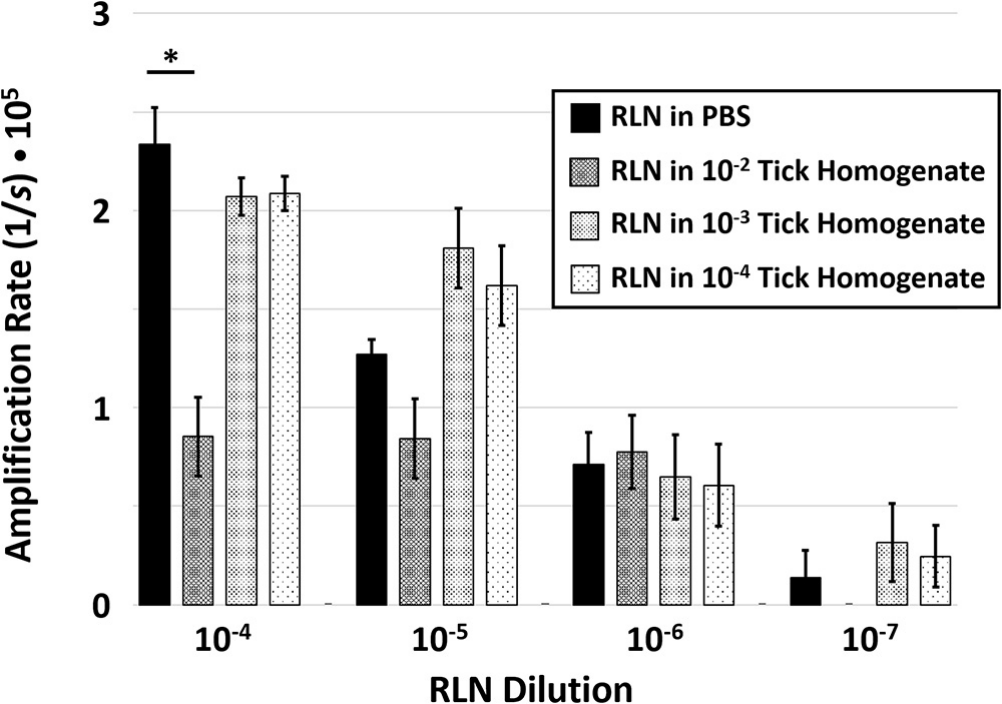
Amplification rates of CWD-positive retropharyngeal lymph node (RLN) dilutions in phosphate-buffered saline (PBS) or tick homogenate dilutions. Amplification rates were calculated as the average inverse of the time (in seconds) to amplification threshold in six replicate wells across two experimental plates. Standard error bars are shown. Using a two-tailed Student’s *t* test, a significant reduction in amplification (*, *P* ≤ 0.01) was observed in the 10^−4^ dilution of RLN in PBS compared to RLN diluted in 10^−2^ tick homogenate.

Pilot experiments found that RLN homogenates prepared in both 10^−3^ and 10^−4^ dilutions of tick homogenate amplified at rates most similar to RLN homogenates prepared in phosphate-buffered saline (PBS) alone. Tick homogenates of the 10^−3^ dilution were selected for primary studies based on this similarity and to avoid sacrificing potential dilutional sensitivity in the 10^−4^ dilution of tick homogenates. Retropharyngeal lymph node dilutions down to 10^−7^ amplified in a 48-h assay, representing the lower limits of detection for our study (Fig. 1).

### RT-QuIC amplification of CWD prions in ticks.

In the primary experiments, results from three amplification plates were discarded due to amplification in a single negative-control replicate (e.g., one of nine replicates from negative-control ticks); samples from these plates were subsequently repeated with no amplification among negative controls. One plate was discarded due to failure of a single positive-control replicate to amplify. Not including these discarded plates, two plates were run for the preliminary optimization experiments, and 10 plates were run in a blind manner using tick samples.

**FIG 2:**
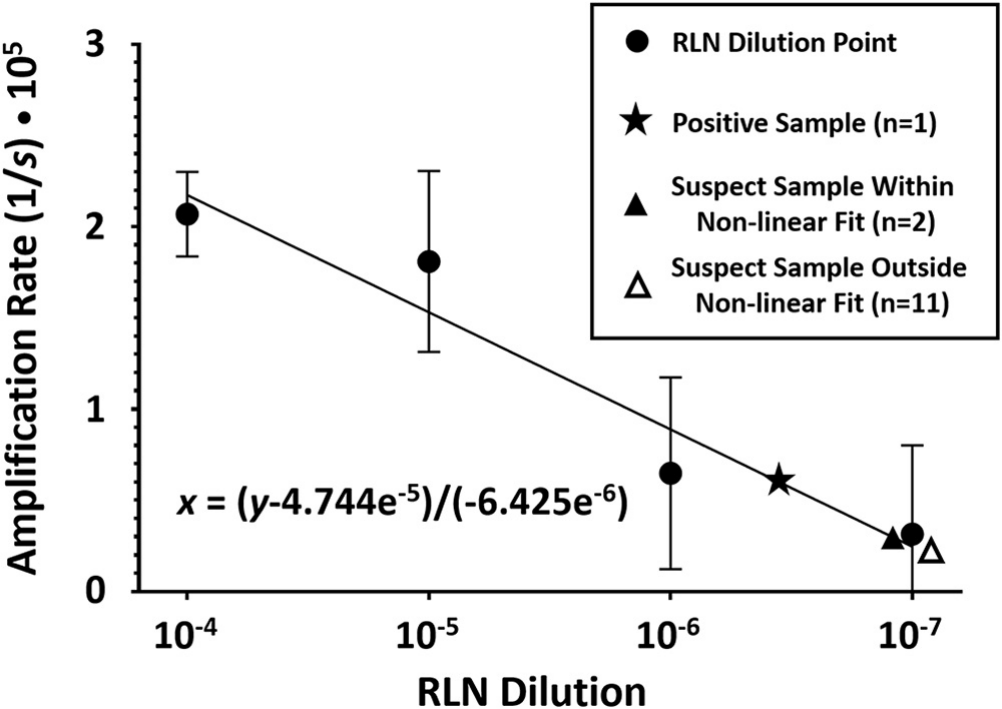
Nonlinear regression fit of data from CWD-positive retropharyngeal lymph node (RLN) dilutions in a 10^−3^ tick homogenate. Amplification rates were calculated as the inverse of the average time (in seconds) to the amplification threshold in six replicate wells across two experimental plates. Data points shown include RLN dilutions with standard error bars as well as a single RT-QuIC-positive sample and suspect samples both within and outside the range of RLN dilutional data. The slope of the nonlinear regression line is indicated as *x* = (*y* − 4.744^−5^)/(−6.425e^−6^).

Among the 136 ticks analyzed from elk, a single positive tick was identified based on amplification in three of six replicates across two experiments performed in a blind manner. This tick was collected from a CWD-positive elk in late-stage symptomatic disease, and the mean amplification rate was approximately equal to a 10^−6.5^ dilution of CWD-positive RLN (Fig. 2). Thirteen ticks demonstrated amplification considered “suspect,” with one or two of six replicates showing amplification, all with mean amplification rates between 10^−6.9^ and 10^−7.2^ RLN dilutional equivalents (e.g., approximating or beyond the lower range of our nonlinear regression fit). Of these suspects, six were from CWD-positive elk in middle stages of disease, while seven were from CWD-negative elk (see Table S1 in the supplemental material). Ticks from CWD-positive elk were significantly more likely to be considered “suspect” than CWD-negative elk using a two-tailed Fisher exact test (6/29 versus 7/106; *P* = 0.0337). No evidence of amplification was observed in ticks from 23/30 CWD-positive elk and 106/113 CWD-negative elk (Table 2).

**TABLE 2:** Summary of RT-QuIC results from ticks collected from CWD-positive and -negative elk^*a*^

Elk CWD status	Total no. of elk	RT-QuIC result for winter ticks		
		No amplification	Suspect	Positive
Early (B or RLN)	5	5	0	0
Middle (B and RLN)	24	18	6	0
Late (B and RLN and symptomatic)	1	0	0	1
Negative	106	99	7	0

a Disease stage estimates were again based on immunohistochemical detection of PrP^CWD^ in the elk’s brain (B) and/or retropharyngeal lymph node (RLN) and clinical signs consistent with late-stage infection, including behavior abnormalities and poor body condition scores.

10.1128/mSphere.00515-21.1 TABLE S1 Summary of raw data collected from RT-QuIC amplification of tick substrates. CWD status is based on clinical and pathological findings. The number of positive replicates indicates the number of replicates crossing a predetermined threshold for amplification, with those samples amplifying in 1 or 2 of 6 replicates considered “suspect” and those amplifying in ≥3 replicates considered positive. The relative rate of amplification is a comparison of the mean rates of amplification, considering both amplifying and nonamplifying replicates, to the standardized control (a 10^−5^ dilution of RLN in a 10^−3^ tick homogenate in PBS). Download Table S1, DOCX file, 0.01 MB. Copyright © 2021 Haley et al. 2021 Haley et al. https://creativecommons.org/licenses/by/4.0/ This content is distributed under the terms of the Creative Commons Attribution 4.0 International license.

## DISCUSSION

With the sharp global decline in reported sheep scrapie and bovine spongiform encephalopathy cases (37, 38), chronic wasting disease of cervids remains one of the most important mammalian prion diseases identified thus far—occurring in both free-ranging and farmed populations of deer and related species across North America and now Scandinavia (39). Over the past 2 decades, much has been learned about the mechanisms of CWD transmission, primarily through the evaluation of blood and other body fluids in multiyear-long cervid or murine bioassay experiments (10, 11, 13, 22, 40–45). More recently, *in vitro* amplification assays, which typically offer results in several days, have sought to supplant bioassay as the dominant testing modality for PrP^CWD^ in various forms of excreta (13–17, 46). These assays have been systematically used to evaluate the onset, duration, and severity of PrP^CWD^ shedding in saliva, urine, and feces. The real-time quaking-induced conversion assay (RT-QuIC) in particular has been shown to be sensitive, specific, and highly reproducible when evaluating biological samples for CWD prions (31, 32, 47). In the present study, we evaluated ticks collected from CWD-negative elk and elk in various stages of CWD ranging from early preclinical infections to late-stage symptomatic disease to better characterize their potential role as vectors of CWD transmission.

We began by roughly optimizing tick homogenate concentrations for use in the RT-QuIC assay by comparing the amplification of PrP^CWD^ from known positive RLN tissue in various dilutions of background tick homogenate. We found that RLN diluted in a 10^−3^ preparation of tick homogenate allowed for amplification of PrP^CWD^ similar to RLN diluted in PBS alone, without potentially sacrificing diluted sensitivity. The comparatively poor amplification observed in the 10^−2^ preparation may have been a result of putative inhibitors common to some sample matrices (16, 17, 48). Though we did not attempt various concentration methods, e.g., iron oxide beads, they may prove helpful in reducing matrix inhibition and improving sensitivity in future experiments (16, 49).

We went on to evaluate ticks collected from 136 elk in a blind manner in triplicate and in two independent experiments. We identified a single tick as RT-QuIC positive based on amplification in three of six replicates; this tick had been collected from an elk in terminal stages of CWD based on clinical and pathological findings. Several ticks from both CWD-negative and CWD-positive elk were found to be RT-QuIC “suspects,” based on amplification in one or two of six replicates. Ticks from CWD-positive elk, specifically those in mid-stage CWD, were significantly more likely to be considered RT-QuIC suspects than those from CWD-negative elk. The rates of amplification observed in suspect cases were at the lower limits of detection for the assay, and the biological relevance of true PrP^CWD^ amplified from RT-QuIC suspect and positive ticks is not known. Importantly, we cannot completely rule out dermal or environmental contamination of the ticks, from dust or excreta for example. We likewise cannot rule out the possibility that ticks from CWD-negative elk which showed amplification had not recently relocated from a CWD-positive elk, though this seems unlikely. We must therefore be cautious not to weigh these findings too heavily, and instead reinforce the importance of adequate negative controls and proper experimental blinding in future prion amplification studies (50).

Admittedly, additional experiments on D. albipictus and other tick species from wapiti, especially those in later stages of CWD, are necessary to further explore the role of ticks as prion reservoirs. Because of suspected differences in CWD pathogenesis between deer species and North American elk, it would also be important to analyze ticks collected from both whitetail and mule deer (51, 52). A recent study in a hamster model of CWD found that the Rocky Mountain wood tick, Dermacentor andersoni, is unlikely to transmit biologically relevant levels of prions to a naive host after consuming a blood meal (53). It is important to highlight that, unlike D. andersoni, the winter ticks evaluated in the present study are one-host ticks (25). These single-host ticks represent ideal targets to assess PrP^CWD^ accumulation, as they had consumed several blood meals prior to collection, yet they are arguably unlikely to transmit infectious agents between hosts in nature. Though they have only rarely been suspected as disease vectors, winter ticks are thought to seek new hosts when feeding is interrupted, for example when removed through grooming or, understandably, following the demise of the host due to, e.g., CWD (25, 26, 29, 54). Vertical transmission of adult *D. andersoni* ticks to neonatal moose calves has also been reported (28). For those reasons, a better understanding of the biological relevance of any detectable PrP^CWD^ in this and other species of ticks is warranted.

In summary, we report that RT-QuIC may serve as a useful tool for evaluating the role of ticks and other insects as reservoirs of PrP^CWD^. Amplifiable levels of PrP^CWD^ in the present study were low, and likely limited to ticks collected from animals in later stages of disease. Additional studies focusing on insect vectors feeding on terminally infected cervids and the biological relevance of any detectable CWD prions in these vectors are warranted to more fully characterize the role of external parasites in prion transmission.

## MATERIALS AND METHODS

### Ethics statement.

The animals from which ticks were collected in this study were handled humanely in accordance with Midwestern University’s Animal Care and Use Committee, approval 2814.

### Elk study population.

The elk involved in the study were part of a private, closed herd living on 3,500 acres of fenced-in land in northwestern Colorado. A thorough description of the study population and area may be found elsewhere (32). Winter ticks were collected from 136 bull and cow elk ranging in age from calves to 14 years. A mixture of 132M/L genotypes were represented among the elk sampled. Postmortem tissues including both retropharyngeal lymph node (RLN) and brain stem at the level of the obex were evaluated for evidence of CWD infection using immunohistochemistry as described previously (31).

### Tick collection and processing.

Adult winter ticks were collected in a clean manner, using single-use gloves, from the pinnae of elk and stored at −80°C for approximately 2 years until processing. At that time, a single tick was recovered from storage, weighed, homogenized in phosphate-buffered saline (PBS) at 1% (wt/vol) using a bead homogenizer and frozen prior to analysis. After thawing, homogenates were further diluted for experimental evaluation as described in the following section. Ticks collected from three elk calves with the 132LL genotype were identified to serve as putative negative controls throughout the study.

### Optimization of the real-time quaking-induced conversion assay (RT-QuIC) for tick substrate.

To find a roughly optimal tick starting dilution without sacrificing dilutional sensitivity, a pilot experiment was conducted to assess the amplification ability of PrP^CWD^ from a known CWD-positive RLN in three dilutions of tick substrate, 1%, 0.1%, and 0.01% (10^−2-4^) tick homogenates prepared in PBS, compared to RLN homogenized in PBS alone. First, RLN from a known CWD-positive whitetail deer was homogenized in PBS at a 10% (10^−1^) (wt/vol) dilution. Aliquots of this RLN preparation were then diluted serially 10-fold from 10^−4^ to 10^−7^ in either PBS or 10^−2^, 10^−3^, or 10^−4^ homogenates of ticks collected from 132LL genotype elk calves. Amplification was performed using a truncated form of recombinant Syrian hamster PrP (SHrPrP, residues 90 to 231) as a conversion substrate. The recombinant SHrPrP was prepared off-site, frozen at −80°C, and thawed slowly just prior to use in each experiment. Two microliters of each of the preparations was added to 98 μl of RT-QuIC master mix (50 mM NaPO_4_, 350 mM NaCl, 1.0 mM EDTA, 10 mM thioflavin T [ThT], and 0.1 mg/ml SHrPrP). Individual sample dilutions were repeated in triplicate, in two separate experiments, in a 96-well, optical-bottom plate, sealed, and incubated in a BMG Labtech Polarstar fluorimeter at 42°C for 48 h (192 cycles, 15 min each) with intermittent shaking. Cycle parameters included 1-min shakes (700 rpm, double orbital pattern) interrupted by 1-min rest periods, with ThT fluorescence measurements (450-nm excitation and 480-nm emission) taken every 15 min with the gain set at 1,800. The relative fluorescence units (RFU) for each triplicate sample were progressively monitored against time with orbital averaging and 20 flashes/well at the 4-mm setting. Time to amplification was determined based on individual replicates crossing an experimental threshold calculated as 10 standard deviations above the mean fluorescence of all sample wells across amplification cycles 2 to 8, as described in previous studies (31, 32, 47).

### RT-QuIC evaluation of ticks collected from elk.

After identifying appropriate dilution levels for tick homogenates and RLN positive controls, study ticks were individually homogenized and evaluated by RT-QuIC using parameters described above. Study plates included (i) positive RLN controls repeated in triplicate, consisting of a 10^−5^ dilution of RLN in a 10^−3^ tick homogenate in PBS, which typically amplified between cycle number 80 to 100; (ii) individual tick samples prepared at a 10^−3^ dilution evaluated in triplicate; (iii) three unique negative controls, each prepared in triplicate (nine total replicates), consisting of tick homogenates from three elk calves with 132LL genotypes, also prepared at a 10^−3^ dilution; and (iv) unspiked SHrPrP, also in triplicate. Tick homogenates were analyzed in a blind manner, without information on source animal CWD status during tick processing, amplification, and data analysis stages, with CWD status revealed only upon completion of the analysis. All samples were repeated in triplicate in two separate experiments for a total of six replicates.

Criteria for identification of positive samples was determined *a priori* and was consistent with previous studies in our laboratories (31, 32, 47). A replicate well was considered positive when the relative fluorescence crossed a predefined threshold as described above. Suspect samples were those which crossed the threshold in fewer than or equal to one-third of all replicates (e.g., one or two replicates out of six). Positive samples were those which crossed the threshold in ≥3 out of 6 replicates. Plates were disqualified if any of the positive-control replicates failed to amplify, or if amplification was observed in any of the various negative-control replicates.

Amplification rates of positive and suspect samples, including both amplifying and nonamplifying replicates, were subsequently compared to a standard curve generated in the preliminary experiment, using RLN serially diluted into a 10^−3^ dilution of tick homogenate.

### Statistical analysis.

In our preliminary experiment, differences between amplification rates assessing RLN dilutions in various tick backgrounds were compared using two-tailed Student’s *t* tests. Approximate RLN dilutional equivalents of RT-QuIC-positive and RT-QuIC suspect ticks were calculated by comparing the mean amplification rate of the six replicates (e.g., both amplifying and nonreactive wells) to a nonlinear fit of data from RLN diluted in 10^−3^ tick homogenate in preliminary tick dilution experiments, developed using GraphPad Prism 8.4.1 software. Comparison of the likelihood of “suspect” amplification occurring in ticks collected from CWD-negative and CWD-positive elk was performed using a two-tailed Fisher exact test.
