# Supplementary material for: Evaluation of Winter Ticks (Dermacentor albipictus) Collected from North American Elk (Cervus canadensis) in an Area of Chronic Wasting Disease Endemicity for Evidence of PrPCWD Amplification Using Real-Time Quaking-Induced Conversion Assay
Source: mSphere. 2021 Aug 4;6(4):e00515-21. doi: 10.1128/mSphere.00515-21 (PMC8386475; doi:10.1128/mSphere.00515-21)
Supplement: TABLE S1 [file msphere.00515-21-st001.docx]

| CWD Status | Elk ID | Number of positive replicates | Relative rate of amplification |
| --- | --- | --- | --- |
| Late Stage | White 17 | 3 | 0.4532 |
| Mid-stage | White 164 | 2 | 0.2202 |
|  | White 108 | 1 | 0.1396 |
|  | 542 | 1 | 0.1296 |
|  | White 44 | 1 | 0.1264 |
|  | 623 | 1 | 0.1030 |
|  | 502 | 1 | 0.0762 |
| CWD-Negative | White 92 | 2 | 0.1862 |
|  | 643 | 1 | 0.1620 |
|  | White 74 | 1 | 0.1515 |
|  | 628 | 1 | 0.1029 |
|  | 663 | 1 | 0.0861 |
|  | White 52 | 1 | 0.0834 |
|  | 506 | 1 | 0.0784 |
